# 4-Amino-*N*-(3-meth­oxy­pyrazin-2-yl)benzene­sulfonamide

**DOI:** 10.1107/S1600536810038158

**Published:** 2010-09-30

**Authors:** Bruno Bruni, Silvia A. Coran, Gianluca Bartolucci, Massimo Di Vaira

**Affiliations:** a Dipartimento di Scienze Farmaceutiche, Universitá di Firenze, Via U. Schiff 6, I-50019 Sesto Fiorentino, Firenze, Italy; b Dipartimento di Chimica, Universitá di Firenze, Via della Lastruccia 3, I-50019 Sesto Fiorentino, Firenze, Italy

## Abstract

The overall mol­ecular geometry of the title compound, C_11_H_12_N_4_O_3_S, is bent, with a dihedral angle of 89.24 (5)° between the best planes through the two aromatic rings. Each mol­ecule behaves as a hydrogen-bond donor toward three different mol­ecules, through its amidic and the two aminic H atoms, and it behaves as a hydrogen-bond acceptor from two other mol­ecules *via* one of its sulfonamidic O atoms. In the crystal, mol­ecules linked by N—H⋯N and N—H⋯O hydrogen bonds form kinked layers parallel to (001), adjacent layers being connected by van der Waals inter­actions.

## Related literature

The title compound is a prolonged-action drug known as sulfameth­oxy­pyrazine or sulfalene, traditionally used for the treatment of urinary tract infections and chronic bronchitis. It is also presently employed in combination with other drugs for the treatment of malaria and other diseases. For the pharmacological applications of the title compound, see: Adam & Hagelnur (2009 ▶); Penali & Jansen (2008 ▶). For the structure of a related anti­cancer drug, see: Liu *et al.* (1994 ▶). For hydrogen-bond motifs, see: Bernstein *et al.* (1995 ▶).
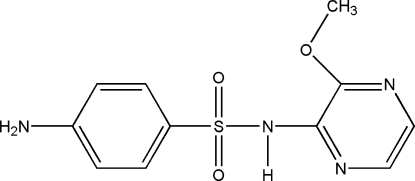


## Experimental

### 

#### Crystal data

C_11_H_12_N_4_O_3_S
*M* *_r_* = 280.31
Orthorhombic, 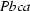

*a* = 10.7589 (2) Å
*b* = 9.5652 (2) Å
*c* = 24.5586 (4) Å
*V* = 2527.35 (8) Å^3^
*Z* = 8
Cu *K*α radiation
μ = 2.40 mm^−1^
*T* = 150 K
0.60 × 0.10 × 0.10 mm

#### Data collection

Oxford Diffraction Xcalibur PX Ultra CCD diffractometer
Absorption correction: multi-scan (*ABSPACK*; Oxford Diffraction, 2006 ▶) *T*_min_ = 0.477, *T*_max_ = 1.000
5771 measured reflections
2373 independent reflections
2103 reflections with *I* > 2σ(*I*)
*R*_int_ = 0.016

#### Refinement

*R*[*F* ^2^ > 2σ(*F* ^2^)] = 0.032
*wR*(*F* ^2^) = 0.103
*S* = 1.14
2373 reflections
184 parameters
H atoms treated by a mixture of independent and constrained refinement
Δρ_max_ = 0.29 e Å^−3^
Δρ_min_ = −0.41 e Å^−3^

### 

Data collection: *CrysAlis PRO CCD* (Oxford Diffraction, 2006 ▶); cell refinement: *CrysAlis PRO CCD*; data reduction: *CrysAlis PRO RED* (Oxford Diffraction, 2006 ▶); program(s) used to solve structure: *SIR2004* (Burla *et al.*, 2005 ▶); program(s) used to refine structure: *SHELXL97* (Sheldrick, 2008 ▶); molecular graphics: *ORTEP-3* (Farrugia, 1997 ▶) and *PLATON* (Spek, 2009 ▶); software used to prepare material for publication: *SHELXL97*, *WinGX* (Farrugia, 1999 ▶) and *PARST* (Nardelli, 1995 ▶).

## Supplementary Material

Crystal structure: contains datablocks global, I. DOI: 10.1107/S1600536810038158/fl2318sup1.cif

Structure factors: contains datablocks I. DOI: 10.1107/S1600536810038158/fl2318Isup2.hkl

Additional supplementary materials:  crystallographic information; 3D view; checkCIF report

## Figures and Tables

**Table 1:** Hydrogen-bond geometry (Å, °)

*D*—H⋯*A*	*D*—H	H⋯*A*	*D*⋯*A*	*D*—H⋯*A*
N3—H3*N*⋯N1^i^	0.85 (2)	2.24 (2)	3.063 (2)	164 (2)
N4—H42⋯O1^ii^	0.91 (3)	2.19 (3)	3.033 (2)	154 (2)
N4—H41⋯O1^iii^	0.90 (3)	2.37 (3)	3.266 (2)	176 (2)

Symmetry codes: (i) 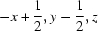; (ii) 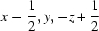; (iii) 
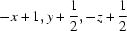
.

**Fig. 1.**
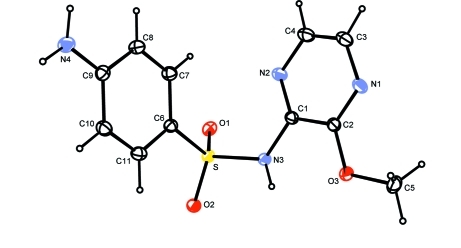
A view of the molecule forming the asymmetric unit of the title compound. Displacement ellipsoids are drawn at the 30% probability level.

**Fig. 2.**
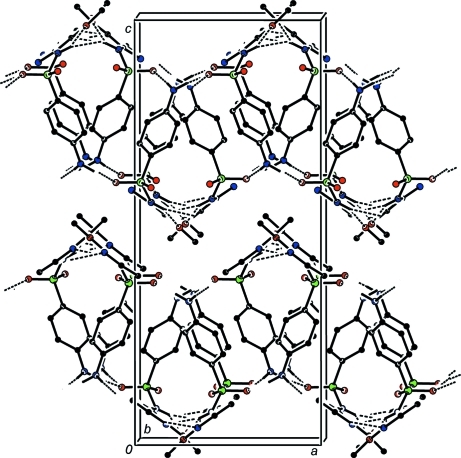
The arrangement of molecules in the structure of I, viewed along the b direction. Only hydrogen atoms involved in the formation of hydrogen bonds are shown for clarity. Hydrogen bonds are denoted by dashed lines.

## supplementary crystallographic information

### Comment

The title compound (I), a drug known as sulfamethoxypyrazine or sulfalene, has been in use for a long time in the treatment of urinary tract infections and chronic bronchitis. Recently, due to the onset of Plasmodium falciparum resistance to many antimalarial drugs (*e.g.* chloroquine), the use of sulfamethoxypyrazine in combination with the dihydrofolate reductase inhibitor pyrimethamine has acquired great importance (Penali & Jansen, 2008). These formulations are also being tested against other deseases, like cutaneous leishmaniasis (Adam & Hagelnur, 2009).

In the structure of I there is one molecule of the title compound in the asymmetric unit of the orthorhombic unit cell. As a consequence of the arrangement of bonds around the sulfur atom, the overall molecular geometry is bent (Fig. 1), with an 89.24 (5) ° angle between the best planes through the two aromatic rings. The amidic N—H bond is almost parallel to the pyrazine plane, the H atom deviating by 0.28 (2) Å from that plane, and the plane of the aminic group is almost parallel to that of the phenyl ring, forming a 12 (2) ° angle with it. Each molecule behaves as a hydrogen bond donor toward three different molecules, through its amidic and the two aminic H atoms, and it behaves as a hydrogen bond acceptor from two other molecules *via* one of its sulfonamidic O atoms. The N—H···N and N—H···O interactions generate, respectively, a C(5) motif and a C_2_^1^(4) binary graph set (Bernstein *et al.*, 1995). Kinked layers of hydrogen–bonded molecules parallel to (001) are formed (Fig. 2), with no additional hydrogen bond interactions between layers. The structure of a somewhat related anticancer drug has been reported (Liu *et al.*, 1994).

### Experimental

Samples of I were kindly provided by SIMS (SIMS srl, Reggello, Firenze, Italy). Crystals of the compound, suitable for X-ray diffraction analysis, were obtained by slow evaporation from 1:1 ethanol:butanol solutions.

### Refinement

In the final refinement cycles non-hydrogen atoms were assigned anisotropic thermal parameters and H atoms had *U*_iso_(H) = 1.2 *U*_eq_(C, *N*), or *U*_iso_(H) = 1.5 *U*_eq_(C) for methyl H atoms. Hydrogen atoms were placed in geometrically generated positions, riding, except for those linked to nitrogen atoms, whose positions were allowed to refine (final values: 0.85 (2) Å for amidic N—H and 0.90 (3)–0.91 (3) Å range for aminic N—H bonds), and for the methyl H atoms, which were refined as part of a group with idealized geometry, freely rotating about the C—C bond. Assigned C—H: aromatic CH 0.950 Å; primary CH 0.952 Å.

### Crystal data

**Table:**

C_11_H_12_N_4_O_3_S	*F*(000) = 1168
*M**_r_* = 280.31	*D*_x_ = 1.473 Mg m^−^^3^
Orthorhombic, *P**b**c**a*	Cu *K*α radiation, λ = 1.54180 Å
Hall symbol: -P 2ac 2ab	Cell parameters from 4106 reflections
*a* = 10.7589 (2) Å	θ = 4.5–71.1°
*b* = 9.5652 (2) Å	µ = 2.40 mm^−^^1^
*c* = 24.5586 (4) Å	*T* = 150 K
*V* = 2527.35 (8) Å^3^	Elongated prism, colourless
*Z* = 8	0.60 × 0.10 × 0.10 mm

### Data collection

**Table:**

Oxford Diffraction Xcalibur PX Ultra CCD diffractometer	2373 independent reflections
Radiation source: fine-focus sealed tube	2103 reflections with *I* > 2σ(*I*)
Oxford Diffraction, Enhance ULTRA assembly	*R*_int_ = 0.016
Detector resolution: 8.1241 pixels mm^-1^	θ_max_ = 71.2°, θ_min_ = 5.5°
ω scans	*h* = −8→12
Absorption correction: multi-scan (ABSPACK; Oxford Diffraction, 2006)	*k* = −7→11
*T*_min_ = 0.477, *T*_max_ = 1.000	*l* = −28→29
5771 measured reflections	

### Refinement

**Table:**

Refinement on *F*^2^	Secondary atom site location: difference Fourier map
Least-squares matrix: full	Hydrogen site location: inferred from neighbouring sites
*R*[*F*^2^ > 2σ(*F*^2^)] = 0.032	H atoms treated by a mixture of independent and constrained refinement
*wR*(*F*^2^) = 0.103	*w* = 1/[σ^2^(*F*_o_^2^) + (0.0536*P*)^2^ + 1.4166*P*] where *P* = (*F*_o_^2^ + 2*F*_c_^2^)/3
*S* = 1.14	(Δ/σ)_max_ = 0.001
2373 reflections	Δρ_max_ = 0.29 e Å^−^^3^
184 parameters	Δρ_min_ = −0.41 e Å^−^^3^
0 restraints	Extinction correction: *SHELXL97* (Sheldrick, 2008), Fc^*^=kFc[1+0.001xFc^2^λ^3^/sin(2θ)]^-1/4^
Primary atom site location: structure-invariant direct methods	Extinction coefficient: 0.00110 (16)

### Special details

**Table:**

Geometry. All s.u.'s (except the s.u. in the dihedral angle between two l.s. planes) are estimated using the full covariance matrix. The cell s.u.'s are taken into account individually in the estimation of s.u.'s in distances, angles and torsion angles; correlations between s.u.'s in cell parameters are only used when they are defined by crystal symmetry. An approximate (isotropic) treatment of cell s.u.'s is used for estimating s.u.'s involving l.s. planes.
Refinement. Refinement of *F*^2^ against ALL reflections. The weighted *R*-factor *wR* and goodness of fit *S* are based on *F*^2^, conventional *R*-factors *R* are based on *F*, with *F* set to zero for negative *F*^2^. The threshold expression of *F*^2^ > 2σ(*F*^2^) is used only for calculating *R*-factors(gt) *etc*. and is not relevant to the choice of reflections for refinement. *R*-factors based on *F*^2^ are statistically about twice as large as those based on *F*, and *R*- factors based on ALL data will be even larger.

### Fractional atomic coordinates and isotropic or equivalent isotropic displacement parameters (Å^2^)

**Table:**

	*x*	*y*	*z*	*U*_iso_*/*U*_eq_	
S	0.45913 (4)	0.21136 (4)	0.376497 (16)	0.01994 (17)	
O1	0.59274 (13)	0.21818 (13)	0.37730 (5)	0.0254 (3)	
O2	0.40093 (12)	0.07918 (13)	0.38690 (5)	0.0263 (3)	
N3	0.40614 (15)	0.31297 (15)	0.42568 (6)	0.0221 (3)	
H3N	0.340 (2)	0.283 (2)	0.4404 (9)	0.026*	
C1	0.42548 (17)	0.45781 (18)	0.42714 (7)	0.0217 (4)	
C2	0.34037 (17)	0.53944 (18)	0.45771 (7)	0.0214 (4)	
N1	0.34155 (15)	0.67589 (16)	0.45622 (6)	0.0258 (4)	
C3	0.43419 (19)	0.7358 (2)	0.42669 (8)	0.0299 (4)	
H3	0.4377	0.8347	0.4239	0.036*	
C4	0.52204 (19)	0.6575 (2)	0.40095 (9)	0.0321 (5)	
H4	0.5885	0.7035	0.3828	0.039*	
N2	0.51757 (14)	0.51529 (17)	0.40040 (7)	0.0272 (4)	
O3	0.25850 (12)	0.46696 (12)	0.48767 (5)	0.0255 (3)	
C5	0.1764 (2)	0.5454 (2)	0.52249 (8)	0.0326 (5)	
H51	0.1171 (12)	0.5941 (15)	0.5008 (3)	0.049*	
H52	0.2235 (7)	0.6108 (14)	0.5432 (5)	0.049*	
H53	0.1343 (12)	0.4832 (9)	0.5465 (5)	0.049*	
C6	0.40661 (16)	0.27630 (17)	0.31414 (7)	0.0209 (4)	
C7	0.47006 (16)	0.3858 (2)	0.28819 (8)	0.0242 (4)	
H7	0.5409	0.4267	0.3049	0.029*	
C8	0.42941 (17)	0.43368 (19)	0.23848 (7)	0.0252 (4)	
H8	0.4723	0.5083	0.2212	0.030*	
C9	0.32573 (16)	0.37426 (18)	0.21290 (7)	0.0230 (4)	
C10	0.26457 (17)	0.2631 (2)	0.23915 (7)	0.0262 (4)	
H10	0.1953	0.2201	0.2220	0.031*	
C11	0.30361 (17)	0.21565 (18)	0.28935 (8)	0.0250 (4)	
H11	0.2603	0.1418	0.3069	0.030*	
N4	0.28793 (16)	0.42195 (19)	0.16322 (6)	0.0282 (4)	
H41	0.321 (2)	0.501 (3)	0.1502 (10)	0.034*	
H42	0.219 (2)	0.387 (2)	0.1469 (10)	0.034*	

### Atomic displacement parameters (Å^2^)

**Table:**

	*U*^11^	*U*^22^	*U*^33^	*U*^12^	*U*^13^	*U*^23^
S	0.0234 (3)	0.0165 (2)	0.0200 (2)	0.00331 (15)	0.00297 (15)	0.00145 (15)
O1	0.0264 (7)	0.0259 (7)	0.0240 (7)	0.0062 (5)	0.0027 (5)	0.0034 (5)
O2	0.0363 (8)	0.0165 (6)	0.0262 (6)	0.0009 (5)	0.0048 (6)	0.0012 (5)
N3	0.0267 (8)	0.0169 (7)	0.0225 (7)	−0.0006 (6)	0.0063 (6)	−0.0002 (6)
C1	0.0259 (9)	0.0183 (8)	0.0209 (8)	−0.0016 (7)	−0.0015 (7)	0.0012 (7)
C2	0.0265 (9)	0.0189 (8)	0.0189 (8)	−0.0013 (7)	−0.0027 (7)	0.0007 (7)
N1	0.0320 (8)	0.0175 (7)	0.0278 (8)	−0.0020 (6)	−0.0023 (6)	−0.0015 (6)
C3	0.0345 (10)	0.0197 (9)	0.0353 (10)	−0.0051 (8)	−0.0028 (8)	−0.0001 (8)
C4	0.0319 (10)	0.0261 (10)	0.0385 (11)	−0.0091 (8)	0.0046 (8)	0.0001 (9)
N2	0.0278 (8)	0.0221 (8)	0.0316 (9)	−0.0051 (6)	0.0046 (7)	−0.0007 (7)
O3	0.0325 (7)	0.0194 (6)	0.0244 (6)	0.0011 (5)	0.0081 (5)	−0.0001 (5)
C5	0.0431 (12)	0.0291 (10)	0.0255 (9)	0.0060 (9)	0.0122 (9)	−0.0003 (8)
C6	0.0233 (9)	0.0178 (8)	0.0216 (9)	0.0022 (6)	0.0021 (7)	0.0015 (7)
C7	0.0220 (9)	0.0251 (9)	0.0255 (9)	−0.0018 (7)	−0.0018 (7)	0.0021 (8)
C8	0.0249 (9)	0.0249 (9)	0.0258 (9)	−0.0021 (7)	0.0008 (7)	0.0048 (8)
C9	0.0241 (9)	0.0228 (9)	0.0222 (8)	0.0029 (7)	0.0010 (7)	−0.0001 (7)
C10	0.0255 (9)	0.0246 (9)	0.0285 (9)	−0.0037 (8)	−0.0027 (8)	−0.0002 (8)
C11	0.0263 (9)	0.0205 (9)	0.0282 (9)	−0.0013 (7)	0.0019 (7)	0.0027 (7)
N4	0.0276 (8)	0.0325 (9)	0.0247 (8)	−0.0050 (7)	−0.0044 (7)	0.0057 (7)

### Geometric parameters (Å, °)

**Table:**

S—O2	1.4338 (13)	C5—H51	0.9522
S—O1	1.4392 (14)	C5—H52	0.9522
S—N3	1.6519 (15)	C5—H53	0.9522
S—C6	1.7466 (17)	C6—C11	1.391 (3)
N3—C1	1.401 (2)	C6—C7	1.403 (3)
N3—H3N	0.85 (2)	C7—C8	1.375 (3)
C1—N2	1.310 (2)	C7—H7	0.9500
C1—C2	1.418 (3)	C8—C9	1.401 (3)
C2—N1	1.306 (2)	C8—H8	0.9500
C2—O3	1.341 (2)	C9—N4	1.365 (2)
N1—C3	1.359 (3)	C9—C10	1.407 (3)
C3—C4	1.361 (3)	C10—C11	1.379 (3)
C3—H3	0.9500	C10—H10	0.9500
C4—N2	1.361 (3)	C11—H11	0.9500
C4—H4	0.9500	N4—H41	0.90 (3)
O3—C5	1.440 (2)	N4—H42	0.91 (3)
			
O2—S—O1	118.28 (8)	H51—C5—H52	109.5
O2—S—N3	103.76 (8)	O3—C5—H53	109.5
O1—S—N3	107.94 (8)	H51—C5—H53	109.5
O2—S—C6	109.19 (8)	H52—C5—H53	109.5
O1—S—C6	108.60 (8)	C11—C6—C7	120.01 (16)
N3—S—C6	108.68 (8)	C11—C6—S	119.54 (13)
C1—N3—S	123.30 (13)	C7—C6—S	120.41 (14)
C1—N3—H3N	116.6 (15)	C8—C7—C6	119.81 (17)
S—N3—H3N	113.7 (15)	C8—C7—H7	120.1
N2—C1—N3	120.97 (16)	C6—C7—H7	120.1
N2—C1—C2	121.51 (16)	C7—C8—C9	121.07 (17)
N3—C1—C2	117.51 (16)	C7—C8—H8	119.5
N1—C2—O3	122.59 (16)	C9—C8—H8	119.5
N1—C2—C1	121.94 (17)	N4—C9—C8	120.18 (17)
O3—C2—C1	115.46 (15)	N4—C9—C10	121.54 (17)
C2—N1—C3	116.31 (17)	C8—C9—C10	118.27 (16)
N1—C3—C4	121.68 (18)	C11—C10—C9	121.07 (17)
N1—C3—H3	119.2	C11—C10—H10	119.5
C4—C3—H3	119.2	C9—C10—H10	119.5
N2—C4—C3	121.98 (18)	C10—C11—C6	119.76 (17)
N2—C4—H4	119.0	C10—C11—H11	120.1
C3—C4—H4	119.0	C6—C11—H11	120.1
C1—N2—C4	116.19 (17)	C9—N4—H41	118.9 (15)
C2—O3—C5	117.34 (14)	C9—N4—H42	121.1 (15)
O3—C5—H51	109.5	H41—N4—H42	118 (2)
O3—C5—H52	109.5		
			
O2—S—N3—C1	−170.84 (15)	C1—C2—O3—C5	175.49 (16)
O1—S—N3—C1	62.86 (16)	O2—S—C6—C11	13.30 (17)
C6—S—N3—C1	−54.74 (17)	O1—S—C6—C11	143.57 (14)
S—N3—C1—N2	−22.2 (2)	N3—S—C6—C11	−99.25 (15)
S—N3—C1—C2	156.57 (13)	O2—S—C6—C7	−164.57 (14)
N2—C1—C2—N1	7.2 (3)	O1—S—C6—C7	−34.30 (17)
N3—C1—C2—N1	−171.57 (16)	N3—S—C6—C7	82.88 (16)
N2—C1—C2—O3	−173.32 (16)	C11—C6—C7—C8	0.6 (3)
N3—C1—C2—O3	7.9 (2)	S—C6—C7—C8	178.47 (14)
O3—C2—N1—C3	176.25 (16)	C6—C7—C8—C9	−0.5 (3)
C1—C2—N1—C3	−4.4 (3)	C7—C8—C9—N4	−179.05 (17)
C2—N1—C3—C4	−1.1 (3)	C7—C8—C9—C10	−0.5 (3)
N1—C3—C4—N2	4.3 (3)	N4—C9—C10—C11	179.95 (17)
N3—C1—N2—C4	174.85 (17)	C8—C9—C10—C11	1.5 (3)
C2—C1—N2—C4	−3.9 (3)	C9—C10—C11—C6	−1.3 (3)
C3—C4—N2—C1	−1.5 (3)	C7—C6—C11—C10	0.3 (3)
N1—C2—O3—C5	−5.1 (2)	S—C6—C11—C10	−177.59 (14)

### Hydrogen-bond geometry (Å, °)

**Table:**

*D*—H···*A*	*D*—H	H···*A*	*D*···*A*	*D*—H···*A*
N3—H3N···N1^i^	0.85 (2)	2.24 (2)	3.063 (2)	164 (2)
N4—H42···O1^ii^	0.91 (3)	2.19 (3)	3.033 (2)	154 (2)
N4—H41···O1^iii^	0.90 (3)	2.37 (3)	3.266 (2)	176 (2)

Symmetry codes: (i) −*x*+1/2, *y*−1/2, *z*; (ii) *x*−1/2, *y*, −*z*+1/2; (iii) −*x*+1, *y*+1/2, −*z*+1/2.
